# Micro-Raman Spectroscopy and Univariate Analysis for Monitoring Disease Follow-Up

**DOI:** 10.3390/s110908309

**Published:** 2011-08-25

**Authors:** Carlo Camerlingo, Ines Delfino, Giuseppe Perna, Vito Capozzi, Maria Lepore

**Affiliations:** 1 CNR (Consiglio Nazionale delle Ricerche), Istituto di Cibernetica “E. Caianiello”, 80078, Pozzuoli, Italy; E-Mail: c.camerlingo@cib.na.cnr.it; 2 Biophysics and Nanoscience Centre, CNISM, Facoltà di Scienze, Università della Tuscia, 01100, Viterbo, Italy; E-Mail: delfino@unitus.it; 3 Dipartimento di Scienze Biomediche, Università di Foggia, 71100, Foggia, Italy; E-Mails: g.perna@unina2.it (G.P.); v.capozzi@unifg.it (V.C.); 4 Dipartimento di Medicina Sperimentale, Seconda Università di Napoli, 81100, Naples, Italy

**Keywords:** oral tissues, Raman microspectroscopy, univariate data analysis, follow-up monitoring

## Abstract

Micro-Raman spectroscopy is a very promising tool for medical applications, thanks to its sensitivity to subtle changes in the chemical and structural characteristics of biological specimens. To fully exploit these promises, building a method of data analysis properly suited for the case under study is crucial. Here, a linear or univariate approach using a R^2^ determination coefficient is proposed for discriminating Raman spectra even with small differences. The validity of the proposed approach has been tested using Raman spectra of high purity glucose solutions collected in the 600 to 1,600 cm^−1^ region and also from solutions with two known solutes at different concentrations. After this validation step, the proposed analysis has been applied to Raman spectra from oral human tissues affected by Pemphigus Vulgaris (PV), a rare life-threatening autoimmune disease, for monitoring disease follow-up. Raman spectra have been obtained in the wavenumber regions from 1,050 to 1,700 cm^−1^ and 2,700 to 3,200 cm^−1^ from tissues of patients at different stages of pathology (active PV, under therapy and PV in remission stage) as confirmed by histopathological and immunofluorescence analysis. Differences in the spectra depending on tissue illness stage have been detected at 1,150–1,250 cm^−1^ (amide III) and 1,420–1,450 cm^−1^ (CH_3_ deformation) regions and around 1,650 cm^−1^ (amide I) and 2,930 cm^−1^ (CH_3_ symmetric stretch). The analysis of tissue Raman spectra by the proposed univariate method has allowed us to effectively differentiate tissues at different stages of pathology.

## 1. Introduction

In the last years micro-Raman spectroscopy [1] has shown to be a very promising tool for specific molecular fingerprinting in medical applications. Its success has been strongly boosted by the development of specific data analysis methods enabling the extraction of the wealth of information embedded in Raman spectra of complex samples, such as human tissues, fluids and humours. When large numbers of patients and data are available multivariate methods have been employed for analysing blood and other human aqueous matrices [2–4] and pathological human tissues [5,6]. Principal Component Analysis (PCA) methods have been able to classify oral tissues into different categories depending on the stage of the specific disease (normal, inflammatory, premalignant and malignant) [7]. When a few samples have to be examined, different approaches for comparing Raman spectra have been developed and applied in order to evaluate the occurrence of changes in signal intensity or in spectrum peak configuration with respect to a reference signal [8–13]. In general, the information has been obtained from the direct comparison of selected band areas [8–10] or of single peak intensities [11,12], relevant to the sample state or configuration. When the spectrum modifications are relatively weak, it can be difficult to quantitatively evaluate the effect of possible changes in state or configuration on small spectral regions, because of the presence of noise, background or spurious signals. A global analysis of the spectrum can provide some advantages in the evaluation of spectral changes, especially when the spectra to be compared have a close configuration. Several correlation methods have been typically employed in these cases [14,15] in order to enhance similarities or correlation in the data sets.

In this paper, a univariate approach is proposed for analyzing Raman spectra when only a few samples are available, as in the case of a rare pathology or in the initial stages of a pilot study. The validation and assessment steps have been performed using controlled samples, consisting of high purity glucose aqueous solutions and aqueous mixtures in which different glucose concentrations are present, together with other substances. After the assessment, the proposed approach has been applied to differentiate oral tissues from patients affected by Pemphigus Vulgaris (PV) at different illness stages in order to monitor disease progression and therapy efficacy. PV is a rare life-threatening autoimmune disease that causes blistering of the skin and oral cavity, being characterized by the disruption of keratinocytes’ adhesion [16,17]. The follow-up monitoring of PV is usually accomplished by means of histological features and immunofluorescence analysis. However, alternative techniques for disease follow-up should be developed to better understand the disease progression and to avoid multiple biopsies.

The proposed approach for spectrum analysis can be considered as an extension of the many methods reported in the literature for quantitatively comparing Raman signals [8–13] and it could be particularly useful for comparing Raman responses from different and independent samples of similar materials, such as tissue biopsies. In spite of their differences due to individual characteristics and history, previous work [5–7] has shown that a basic common profile may be expected for Raman responses from tissues of subjects in the same state of pathology. The method here proposed permits one to find out the similarities in the spectra and to discriminate the spectra exhibiting possible anomalies, thus providing a powerful tool for the analysis of spectra with small differences.

## 2. Experimental Section

### 2.1. Sample Preparation and Data Acquisition

Glucose aqueous solutions and aqueous mixed solutions of glucose and artificial sweeteners were used as control samples. High purity glucose powder (>99%) was purchased from Riedel de Haën AG (Buchs, Switzerland) and used without any further treatment. The main components of the commercial sweetener here used were sorbitol, mannitol, fructose and saccharin sodium as reported on the product’s label. A proper amount of glucose powder was dissolved in distilled water to obtain glucose concentrations, [Glu], in the 25 mM–1,050 mM range. Glucose and commercial sweetener were also dissolved in distilled water for preparing six samples with different relative concentrations as indicated in Table 1.

Biopsies of oral tissues from patients with histological and immunofluorescence confirmed PV were used as complex samples. Patients (two subjects) with detectable titres of anti-Inter Cellular Substance Immunoglobuline G (ICS IgG) and at least two oral/skin lesions were considered as “active PV”, whereas those taking immunosuppressive agents at the time of biopsy collection were referred to as “PV in therapy” (two subjects). Patients having no more clinical evidence of blisters and ICS titres <1:40 at the end of the therapy were considered as “PV in remission” (recovered—two patients). Informed consents were obtained from all participants prior to biopsy. Two oral biopsies were obtained from each patient: one was used for direct immunofluorescence tests, and the other for Raman investigations. Samples were put in ice-cold saline immediately after removal and used immediately. When storage was necessary, samples were frozen at liquid nitrogen and stored at −80 °C. They were taken out and passively thawed immediately before recording the spectra. Such a procedure had been shown to not provoke changes in the Raman spectra of tissues [18].

Samples were examined using a micro-Raman spectrometer equipped with a confocal inverted microscope (Horiba-Jobin Yvon, Jongjumeau, France) with a grating of 1,800 grooves/mm, a liquid nitrogen cooled CCD detector (1,024 × 256 chip size) and a 17 mW He-Ne laser operating at 633 nm. The laser light was focused on the sample surface by means of a 50× optical objective (Olympus MPLAN 50×/0.75). The laser excitation spot has dimension of about 20 μm and the actual analyzed area is estimated in about 15 μm^2^ for the microscope configuration adopted with a laser power at sample level equal to few mW. The micro-Raman spectra were detected in the 600–1,600, 1,000–1,700 and 2,700–3,200 cm^−1^ wavenumber shift regions. The spectra were obtained using accumulation times of 600 seconds. For each samples measurements were performed three times on different sites obtaining a total of one hundred files.

Spectra were preliminarily analyzed using the application routines provided by the software package (“SpectraMax_TM_ Software” User Guide, Jobin-Yvon Inc., Edison, NJ, USA) controlling the whole data acquisition system. The spectra typically show a smeared background signal, with intensity of order of about 80% of the whole average intensity. A baseline correction program from the data acquisition software is used. The procedure consists in fixing some points of the spectra (in our case four points) and to subtract to the experimental data the cubic curve obtained by the fit of this fixed points. Typically the background level is weakly dependent on the wavenumber position, with respect to the Raman component of the signal, and this operation, in spite of its intrinsic arbitrariness weakly influences the information and repeatability of the process. No further treatment has been done before the comparison of the data by means of linear regression.

For signal interpretation the complex spectra were also analyzed in terms of convoluted Lorentzian shaped vibration modes [19,20]. Peaks constituting the spectrum were manually selected in order to define the starting conditions for a best-fit procedure. The best-fit procedure was then performed to determine convolution peaks with optimised intensity, position and width. Its performance was evaluated by means of the χ^2^ parameter defined, as usual, by the following formula:

(1) $$\chi^{2}=\frac{\sum_{i=0}^{n}{\left(\frac{{\mathrm{Actual}}_{i}-{\mathrm{Calculated}}_{i}}{\mathrm{RMSNoise}}\right)}^{2}}{\left(n-f\right)}$$

where the *Actual* and *Calculated* are the measured and calculated data, respectively, *RMSNoise* is the estimated Root Mean Square noise in the *Actual* data over the fitted region, *n* is to the number of data points in the fitted region and *f* refers to the total number of variables from the peak and baseline functions. Thus, *n-f* is the number of degrees of freedom. The Levenberg-Marquardt algorithm was employed to adjust every variable for each peak in an attempt to minimize the χ^2^ parameter. This procedure was extremely useful for correctly determining the peak positions, widths, heights and areas of a set of overlapping peaks.

### 2.2. Univariate Data Analysis

For each wavenumber point, Raman intensity of the spectrum (*y_i_* variable) is compared with the corresponding value of a reference spectrum (*x_i_* variable), referring to a sample in pristine condition or to a solution with known concentration. In order to overcome constraints related to the possible different scale of the experimental data, the data set is directly correlated with a linear scaling of the reference signal. This means to do a linear regression or univariate analysis of the data. The basic assumption is that the spectrum Y (containing the n data *y_i_*) is a linear function of the reference spectrum X (containing the n data *x_i_*). All variations with respect to the reference signal are considered as a perturbation term *ε*_i_:

(2) $$y_{i}={\mathrm{mx}}_{i}+p+{ɛ}_{i}$$

If no structural change occurs in the substance generating the Raman spectra, the term *ɛ_i_* depends only on the experimental conditions and we can assume that it has random values that follow a Gaussian distribution, with mean 0 and variance σ_x_, which does not depend on the index *i* [19]. This assumption implies that the *y_i_* are also normally distributed, with σ_y_ = σ_x_. Regression analysis allows to determine, by means of a least square fit of data *y_i_* with respect to *x_i_*, the parameters *m* and *p* and to identify the linear dependence. A parameter useful to check how well a regression equation fits the data is the sample coefficient of determination, R^2^ [14, 21]. It is defined as:

(3) $$R^{2}=1-\frac{\sum {\left[y_{i}-\left({\mathrm{mx}}_{i}+p\right)\right]}^{2}}{\sum {\left[y_{i}-\bar{y}\right]}^{2}}$$

where *ȳ* is the average value of vector Y.

R^2^ ranges from 0 for uncorrelated data, to 1 for perfect linear dependence. When the error *ɛ* is purely random, the value of R*^2^* is equivalent to the square of the covariance *r_yŷ_*, calculated for the vector Y and the prediction vector Ŷ having components *ŷ_i_* = *mx_i_ + p*, which is defined as [14,19]:

(4) ryy^=∑(yi−y¯)(y^i−y^¯)[∑(yi−y¯)2]12[∑(y^i−y^¯)2]12

R^2^ is thus an index of the correlation between the considered set of data and the linear dependence on the reference signal, as we have postulated. The linear regression was evaluated on a number of points ranging between 500 and 700. Due to the high number of points considered, the values reported for R^2^ in the next paragraph imply a high level of significance.

## 3. Results and Discussion

**Figure 1.**
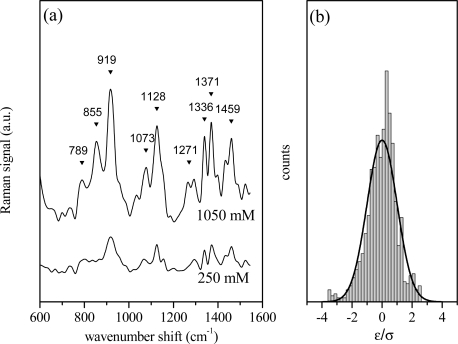
**(a)** Raman spectra (600–1,600 cm^−1^ spectral region) of aqueous glucose solutions at two different glucose concentrations (1,050 mM and 250 mM). Different arbitrary units are used for the vertical axis of the two spectra. The attributions of the main peaks are the following: 789 cm^−1^: α—anomer; 855 cm^−1^: α—anomer; 919 cm^−1^: α—anomer; 1,073 cm^−1^: β—anomer; 1,128 cm^−1^: β—anomer; 1,271 cm^−1^: glucose; 1,336 cm^−1^: α—anomer; 1,371 cm^−1^: α—anomer; 1,459 cm^−1^: glucose [22–24]. **(b)** Histogram of the ɛ_i_ values (see text). The residual values of the linear regression of the two spectra have a Gaussian dependence.

The above described univariate or linear regression approach has been firstly applied to aqueous glucose solutions to simulate an experimental situation in which Raman spectra were obtained from different samples with no structural differences. In Figure 1a the spectra of two glucose solutions at nominal concentrations c_x_ = 1,050 mM (reference data, X) and c_y_ = 250 mM (Y data) are shown. The spectra have been analyzed in terms of Lorentzian shaped vibration modes as reported in previous section and in refs. [19,20]. The displayed interval represents the fingerprint region and is the most significant for the investigated samples. The presence of the characteristic bands of the α anomer at 789, 855, 919, 1,336 and 1,371 cm^−1^ and those of β anomer at 1,073, and 1,128 cm^−1^ indicates that glucose in solution is a mixture of the two anomers with s predominance of the β configuration, as indicated by their relative intensities [22,23]. The peaks at 1,401 and 1,430 cm^−1^ can also be attributed to glucose, as reported in Reference [24]. The contributions at 968, 1,036 and 1,524 cm^−1^ can be related to some impurities present even in the high purity glucose powder (>99%) employed in the present experiments. The histogram of the residual values (*ɛ_i_* = *y_i_* − *mx_i_* − *p*) normalized to the value of standard deviation σ_y_ is shown in Figure 1b. As is evident, the distribution follows a Gaussian dependence, according to the assumption of our hypothesis. In this case, R^2^ has a quite high value (R^2^ = 0.910), close to 1, indicating a good correlation between the two Raman spectra, as expected. In fact, by scrutinizing Figure 1(a), it comes clear that the two spectra differ in intensities and noise characteristics, due to the different glucose content, but the main features are preserved. The differences between the spectra slightly affect the R^2^ value calculated with respect to the spectrum of the solution at the highest glucose concentration (1,050 mM), which has been selected as reference data. R^2^ high value indicates that the spectra are very similar.

**Figure 2.**
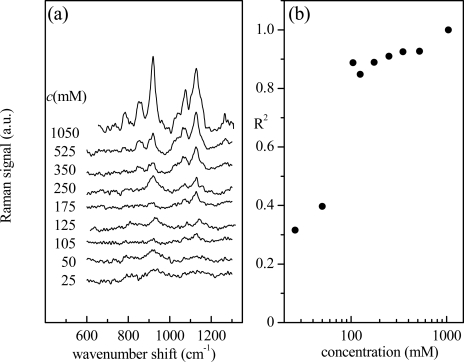
**(a)** Raman spectra (600–1,300 cm^−1^ spectral region) of aqueous glucose solutions at different glucose concentrations (range: 25–1,050 mM). **(b)** R^2^ determination coefficient (95% confidence level) resulting from the linear regression of Raman spectra from glucose solutions in water as a function of the nominal concentration. The analysis has been performed by considering the spectrum from the solution with the highest concentration (1,050 mM) as reference.

This approach can be extended to evaluate, in a simple way, the spectrum similarity for all the investigated aqueous glucose solutions (with glucose concentration ranging from 25 to 1,050 mM). The corresponding spectra, acquired under the same experimental conditions, are shown in Figure 2(a). These spectra are obviously characterized by the spectral features already described for Figure 1(a). As expected, they have similar main characteristics even though intensity and noise contributions are different. The value of R^2^, obtained by comparison to the reference spectrum, as a function of glucose nominal concentration is shown in Figure 2(b). For concentrations lower than 125 mM, the Raman signal from glucose becomes comparable to that generated by noise and the correlation with the reference signal decreases abruptly.

**Figure 3.**
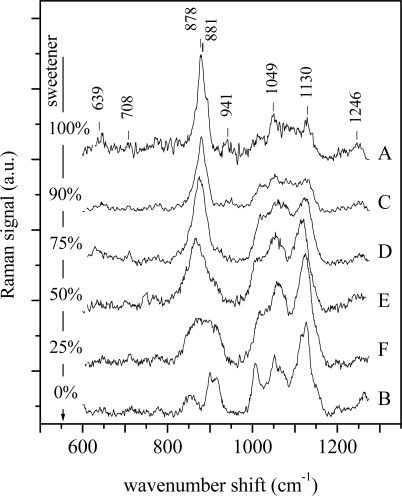
Raman spectra of aqueous solutions of bare sweetener (A), of glucose (B) and of sweetener/glucose mixtures with decreasing weight fraction of sweetener of 90%, 75%, 50% and 25% (C, D, E and F, respectively). The attribution of the main peaks for sample A spectrum are the following: 639 cm^−1^: fructose; 708 cm^−1^: fructose; 878 cm^−1^: mannitol and sorbitol; 881 cm^−1^: fructose; 941 cm^−1^: sorbitol and mannitol; 1,049 cm^−1^: sorbitol and mannitol; 1,130 cm^−1^: mannitol; 1,246 cm^−1^: mannitol [25–27].

To further test this univariate approach, it has been applied to the spectra from the second set of control samples. These solutions, composed of glucose and artificial sweetener in distilled water, were prepared to simulate samples with different chemical compositions. The experimental spectra are shown in Figure 3. Also in this case the spectra of samples have been analyzed as previously reported. In particular for sample A (pure sweetener) the peaks at 639 cm^−1^, 708 cm^−1^ and 881 cm^−1^ are assigned to fructose, the peaks at 878 cm^−1^, 941 cm^−1^ and 1,049 cm^−1^ can be ascribed to mannitol and sorbitol, while the other two peaks at 1,130 cm^−1^ and 1,246 cm^−1^ are due to the contribution of mannitol [25–27].

**Figure 4.**
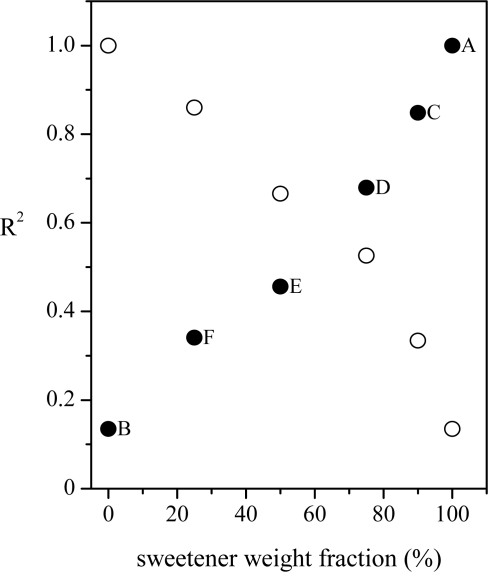
R^2^ coefficient values (95% confidence level) for the linear regression of Raman spectra of different glucose/sweetener mixtures as a function of the sweetener weight fraction (in %). Filled dots represent the values obtained by using the bare sweetener signal (from sample A) as reference. With empty dots the results obtained by using the pure glucose spectrum (from sample B) as reference are shown.

By inspecting the figure, it comes clear that the spectra of C, D, E and F samples are a superposition of the spectra of pure sweetener (sample A) and pure glucose (sample B), as expected. Also in this case the linear regression approach has been applied by considering, in turn, both spectra of samples A and B as reference, the results being shown in Figure 4. In both the cases the approach seems to work well. In fact, when the pure glucose solution is used as reference, the R^2^ coefficient reaches its highest value (close to 1, meaning high similarity) when the reference spectrum is compared to that of pure glucose solution and has decreasing value when the glucose content decreases. The lowest similarity (lowest R^2^ value, close to 0) is obtained when the spectrum of the pure sweetener solution is analyzed. Specular results have been obtained when the spectrum of the pure sweetener is used as reference. In that case, the highest correlation (and, thus, the highest similarity) is observed when the spectrum of the pure sweetener is considered, while R^2^ assumes a value close to 0 when the spectrum of pure glucose is compared with the reference. It’s worth noting that the R^2^ values cover all the available range (0–1) and that there is a clear dependence on the sample chemical compositions, thus suggesting the ability of the method to discriminate between spectra, even when the differences are small.

These encouraging results with samples of increasing degree of complexity have boosted us to apply the proposed approach to very complex Raman spectra as those from oral tissues from patients affected by PV at different illness stages that are characterized by different chemical and structural properties [16,17] in order to monitor the disease evolution and the therapy efficacy.

**Figure 5.**
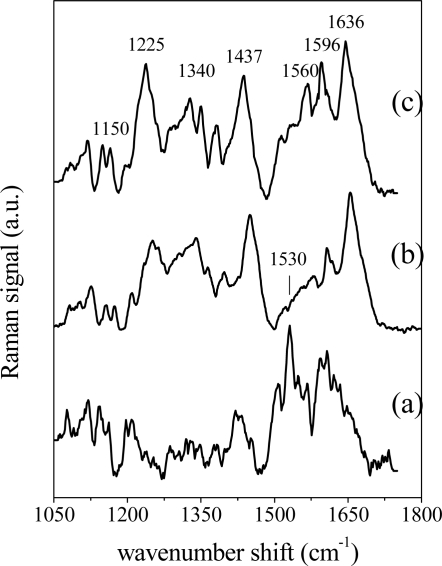
Raman spectra of oral tissue biopsies from PV affected patients **(a)**, patients under drug therapy **(b)** and patients in the remission stage of illness **(c)** in the wavenumber range 1,050–1,700 cm^−1^.

Representative Raman spectra (1,050–1,700 cm^−1^ spectral region) of oral biopsies from PV affected patients, from patients under therapy and from patients in a remission stage of illness (recovered) are shown in Figure 5(a,b,c), respectively. Main structures around 1,150 cm^−1^ (C-C stretch and COH deformation), 1,225 cm^−1^ (amide III), 1,340 cm^−1^, 1,430–1,450 cm^−1^ (CH_3_ deformation), 1,560 cm^−1^ and 1,636 cm^−1^ (amide I) are observed in all the spectra (see references [28] and [29] for attributions), their shape and intensity may be dependent on tissue illness stage. For instance, in Figure 5(a) very poor structures in the initial 1,250–1,400 cm^−1^ region are found in the spectrum of PV active tissues. This finding is related to the decreasing content of phospholipids (whose Raman features are known to fall in this region) in diseased tissues compared to normal ones [7]. On the other hand, the region around 1,500–1,650 cm^−1^ (Figure 5(a)—PV active) is rich in structural features, due to major contributions from proteins [7,29]. These findings are similar to previous literature reports about Raman spectroscopy on normal and cancerous tissues. Pathologies generally do not cause the appearance and the disappearance of peaks and bands in tissue Raman spectra, rather they cause the modification of specific Raman feature structures [7,18,29]. In particular, the outlined changes confirm that the spectrum of normal oral tissue is closer to the lipid spectrum, while the spectra of malignant samples are rich in features assignable to proteins [7,18,30].

**Figure 6.**
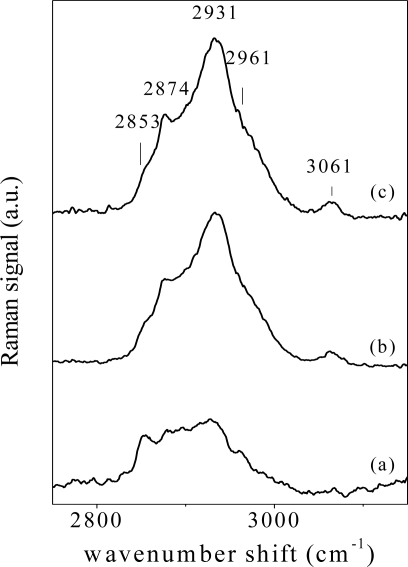
Raman spectra of oral tissue biopsies from PV affected patients **(a)**, patients under drug therapy **(b)** and patients in the remission stage of illness (recovered) **(c)** in the wavenumber range 2,700–3,200 cm^−1^.

In Figure 6, representative spectra (2,700–3,200 cm^−1^ wavenumber region) of tissues from oral biopsies from affected PV patients, from patients under therapy and from patients in remission stage of illness (recovered) are shown in Figure 6(a,b,c) respectively. Samples from two different subjects have been considered for each physiological state. Changes of main components at 2,853 cm^−1^ (CH_2_ symmetric stretch), 2,874 cm^−1^ (CH stretch), 2,931 cm^−1^ (CH_3_ symmetric stretch), 2,961 cm^−1^ (CH_3_ asymmetric stretch) and 3,061 cm^−1^ (CH olefinic stretch) can be easily observed, depending on tissue illness stage. These structures are generally attributed to lipid contents and can be sensitive to tissue biochemical and structural changes [29].

**Figure 7.**
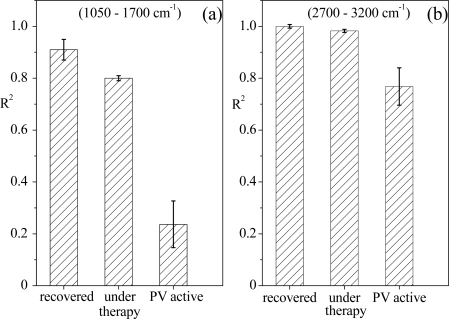
R^2^ values (95% confidence level) resulting from linear regression analysis of Raman spectra relative to oral biopsies from (recovered) patientssin the remission stage of illness, from patients under drug therapy and from affected PV patients in the wavenumber 1,050–1,700 cm^−1^ region **(a)** and 2,700–3,200 cm^−1^ region **(b)**. Columns indicate the mean of R^2^ values. The vertical bars on the top of columns indicate the R^2^ variability range.

The univariate approach has been applied to the oral tissue spectra, considering the two different wavenumber ranges. By taking one spectrum from tissues of recovered patients as reference, the R^2^ determination coefficient values have been obtained and are reported in Figure 7 for the two examined spectral ranges (similar results were obtained for whatever spectrum of recovered patients we selected).

For both the spectral regions, the R^2^ parameter has the same behaviour, even though for the 1,050–1,700 cm^−1^ region the trend is more evident. In particular, when spectra acquired from tissues at the same illness stage are compared, the R^2^ coefficient is larger than 0.9, confirming that in spite of their differences due to individual characteristics and history, the Raman responses of tissues of subjects in the same state of pathology show a basic common profile, as reported in [5–7]. Since the spectra of samples corresponding to different illness stages are different from that of the reference, the R^2^ coefficient values decrease and in particular become very low for the spectra of tissue of PV active patients. Hence, it is evident that this univariate analysis may provide quantitative information on differences among tissues from patients at different stages of illness in the two wavenumber ranges under investigation, in agreement with our previous results on serum samples [31].

Moreover, the present results confirm the possibility of also using high wavenumber ranges for tissue characterization of oral tissues and for “*in vivo*” applications [32–35]. In fact, the use of Raman spectroscopy in the fingerprint spectral region (800–1,800 cm^−1^) in combination with fiber optics is not easy due to the strong background signal generated in the optical fiber. The necessary suppression of this background signal leads to complicated fiber optic probe designs. As a consequence, probes are relatively large and inflexible, expensive, and it is difficult to achieve reproducible performance with them. In contrast, the high wavenumber spectral region from 2,500 to 3,800 cm^−1^ is virtually free of signal contributions from the fused-silica-based optical fibers. This provides a major advantage in that fiber optic probes for *in-vivo* applications can have very simple designs.

## 4. Conclusions

The proposed univariate method, based on the calculation of the R^2^ determination coefficient, has been proven to be able to quantitatively discriminate complex Raman spectra obtained from human oral tissues depending on their illness stage in order to monitor disease evolution and therapy, and also when a small number of spectra is available as for a rare pathology like PV. This ability has been demonstrated for low and high wavenumber spectral regions. It is worthwhile to mention that the use of high wavenumber spectral region could reinforce the potential of Raman spectroscopy for “*in vivo*” applications. This approach can be the starting point for a systematic “*in vivo*” investigation over a wide number of clinical cases to statistically confirm and to fully assess the reliability of the proposed technique for disease follow-up monitoring.

## Figures and Tables

**Table 1.** Composition of mixed solutions. Details on the used commercial sweetener available on request.

**Sample**	**Sweetener weight fraction (%)**	**Glucose weight fraction (%)**

**A**	100	0
**B**	0	100
**C**	90	10
**D**	75	25
**E**	50	50
**F**	25	75
